# Enhancing neutralizing activity against influenza H1N1/PR8 by engineering a single-domain VL-M2 specific into a bivalent form

**DOI:** 10.1371/journal.pone.0273934

**Published:** 2022-08-31

**Authors:** Phuong Thi Hoang, Quynh Xuan Thi Luong, Seungchan Cho, Yongjun Lee, Kyungho Na, Ramadhani Qurrota Ayun, Thuy Thi Bich Vo, Taehyun Kim, Sukchan Lee

**Affiliations:** 1 Department of Integrative Biotechnology, Sungkyunkwan University, Suwon, Republic of Korea; 2 Daesang Cellgene, Giheung-gu, Yongin-si, Gyeonggi-do, Republic of Korea; 3 Novelgen Co., Ltd., R&D center, Yeongtong-gu, Suwon-si, Gyeonggi-do, Republic of Korea; University of South Dakota, UNITED STATES

## Abstract

Flu disease, with high mortality and morbidity, is caused by the influenza virus. Influenza infections are most effectively prevented through vaccination, but it requires annual reformulation due to the antigenic shift or drift of hemagglutinin and neuraminidase proteins. Increasing resistance to available anti-influenza drugs was also recently reported. The M2 surface protein of the influenza virus is an attractive target for universal vaccine development as it is highly conserved and multifunctional throughout the viral life cycle. This study aimed to discover a single-chain variable fragment (scFv) targeting the M2 protein of influenza A H1N1/PR8, showing neutralizing activity through plaque inhibition in virus replication. Several candidates were isolated using bio-panning, including scFv and single-domain V_L_ target M2 protein, which was displayed on the yeast surface. The scFv/V_L_ proteins were obtained with high yield and high purity through soluble expression in *E*. *coli* BL21 (DE3) pLysE strains. A single-domain V_L_-M2-specific antibody, NVLM10, exhibited the highest binding affinity to influenza virions and was engineered into a bivalent format (NVL2M10) to improve antigen binding. Both antibodies inhibited virus replication in a dose-dependent manner, determined using plaque reduction- and immunocytochemistry assays. Furthermore, bivalent anti-M2 single-domain V_L_ antibodies significantly reduced the plaque number and viral HA protein intensity as well as viral genome (*HA* and *NP*) compared to the monovalent single-domain V_L_ antibodies. This suggests that mono- or bivalent single-domain V_L_ antibodies can exhibit neutralizing activity against influenza virus A, as determined through binding to virus particle activity.

## Introduction

Human seasonal influenza viruses cause approximately 3 to 5 million severe flu cases and 290,000 to 650,000 annual deaths worldwide [1]. The CDC estimates that approximately 24 to 45 million illnesses, 280,000 to 810,000 hospitalizations, and 220,00 to 610,00 deaths have occurred in the United States since 2015 [2]. The main seasonal influenza viruses circulating in humans are the influenza A (subtype A (H1N1) and A (H3N2), and influenza B (B/Yamagata or B/Victoria lineages) viruses. Influenza A and B viruses belonging to the *Orthomyxoviridae* family are segmented negative-stranded RNA viruses. There are eight viral RNA gene segments encoded all viral proteins [3]. Hemagglutinin (HA) and neuraminidase (NA) are major surface proteins associated with virus attachment and release in the virus cycle, while the M2 protein is less common and functions as a proton pump [4, 5]. Vaccination is the most effective method to prevent the infection. However, a yearly flu vaccine is recommended because of the antigenic shift or drift of the viral HA and NA proteins. A quadrivalent influenza vaccine was recently developed and approved against four circulating influenza viruses [2]. Current antiviral influenza drugs have two major classes based on M2 ion channel inhibitors (amantadine and rimantadine) and NA inhibitors (oseltamivir, zanamivir, and peramivir) [6]. However, many drug-resistant strains have been identified and reviewed in detail [7, 8], prompting the development of novel therapeutic strategies against influenza infection.

Antibody-based therapy is a promising alternative for the development of a universal influenza vaccine and novel antiviral drugs. Monoclonal antibodies (mAbs) can be engineered into smaller sizes in many formats, such as antigen binding fragments (Fabs), single-chain variable fragments consisting of variable regions of heavy and light chains connected by a flexible peptide linker, and single-domain V_H_ or V_L_ Abs. These smaller entities can be produced in bacteria with high solubility and retain the antigen-binding properties of parental mAbs, resulting in numerous medical and diagnostic applications [9–11]. *Biswas et al*. reviewed valuable, broadly neutralizing monoclonal antibodies (bnAbs) against the influenza virus, including modifications using variable domains of heavy chain only antibodies (V_H_H) and multidomain antibodies [12].

The M2 protein is also a potential target for monoclonal antibody-based treatment of influenza [13]. M2, which is a transmembrane protein (97 amino acids), includes three parts: the extracellular N-terminal domain (M2e-23 aa), the transmembrane domain (TMdomain-22 aa), and the intracellular C-terminal domain (51 aa) [14]. The M2 protein performs multiple important functions in the influenza virus lifecycle. First, during virion entry into the host cell, M2 acts as an ion channel and becomes activated to weaken the interaction of viral ribonucleoprotein complexes with M1, and to initiate HA protein membrane fusion to release the free viral ribonucleoprotein complexes into the cytosol [15–17]. Furthermore, M2 is synthesized in the Golgi apparatus and prevents new HA precursors from changing the conformation, which is induced by the acidic environment in the trans-Golgi network [18]. Independent of the ion channel activity, the M2 protein also hinders autophagosome degradation, which are produced following viral infection of human lung epithelial cells [19]. M2 also participates in virion assembly and virus budding [20, 21]. Moreover, the M2e sequence is highly conserved among all known human influenza A viruses, which is key to the development of universal vaccines [22–24]. mAbs against M2e (14C2) have prevented viral replication of some influenza A strains *in vitro* and reduced lung virus titers in mice [25, 26]. Various M2e mAbs have been developed that demonstrate prophylactic and therapeutic activity against the influenza virus [27–32]. The preservation of these mAbs was mainly mediated through antibody-dependent cell-mediated cytotoxicity (ADCC) and complement-dependent cytotoxicity (CDC), rather than neutralizing virions through the M2 ion channels [27, 33–35]. An earlier study reported the novel mechanism of a non-neutralizing mAb, rM2ss23, in preventing virus budding and release from infected cells, depending on the influenza virus strains, by cross-linking the surface M2 molecules and affecting the HA-M2 association [31].

In this study, we used bio-panning method to isolate several candidates from human scFv-Tomlinson I+J libraries, including the single-chain variable fragment (scFv) and a single-domain V_L_ specific to the M2 protein of influenza H1N1/PR8 strains, which were expressed on a yeast surface display (YSD) system. The candidates exhibited a binding affinity for influenza virions. Single-domain M2-V_L_ (NVLM10), which showed the highest affinity, was engineered into a bivalent format (NVL2M10) that enhanced the binding affinity to the influenza particles. The binding strength of bivalent M2-V_L_ (NVL2M10) to influenza virus particles resulted in a dose-dependent reduction in plaque number, indicating neutralizing activity.

## Materials and methods

### Construction of antigen expression plasmids using a YSD system

To optimize antigen expression in yeast, one N-glycosylation site of the M2 protein sequence of influenza virus A H1N1/PR8 (288 bp) (Accession number AY768951.1) was changed from asparagine (Asn-N) to glutamine (Gln-Q) (N20Q) before synthesis. The DNA fragment was amplified using PCR and cloned between the *NheI* and *BamHI* (NEB, USA) sites of a pCTCON plasmid (ampicillin-resistant) for YSD (M2::pCTCON). The M2::pCTCON plasmid was transformed into *Saccharomyces cerevisiae* EBY100 competent cells, prepared according to the Clontech manual (Clontech, Japan) as previously described [36]. Briefly, EBY100 yeast was freshly prepared in yeast-peptone-glucose media at 0.4 OD_600_ and made competent by chemical solution (1 M Sorbitol/1 mM CaCl_2_, 0.1 M LiAc/10 mM DTT). The antigen M2::pCTCON plasmids were transformed to the competent yeast in ice-cold 1 M Sorbitol/1 mM CaCl_2_ using electroporation (BioRad, USA). The electroporated yeast cells were added to yeast-peptone-glucose media containing 1 M sorbitol before plating on selective SD media (Clontech, Japan) without tryptophan supplementation and incubated at 28°C for 3 days. The yeast was cultured in the selective media and induced to express in SGCAA media containing 2% galactose.

### YSD expression analysis

The M2 antigen expressed on the yeast surface (M2::YSD) was analyzed using western blotting. Briefly, yeast cells were treated with a protein sample loading buffer and denatured by heating. The supernatants were loaded into 12% polyacrylamide gel electrophoresis (PAGE) wells and transferred to a nitrocellulose membrane. The membranes were blocked in 5% skim milk and Tris-buffered saline containing 0.1% (v/v) Tween 20 (TBS-T). Influenza A M2 polyclonal antibodies (Invitrogen, USA) were used at a 1:5000 dilution as primary antibodies and detected using goat anti-rabbit IgG-HRP conjugated secondary antibodies (Invitrogen, USA).

M2::YSD cells were resuspended in PBS to confirm expression using enzyme-linked immunosorbent assay (ELISA). A total of 100 μL of the yeast suspension at an OD of range 0.4–0.6 was coated onto a maxibinding immunoplate (SPL Life Sciences, Republic of Korea) at 4°C and incubated in a humid box overnight. EBY100 yeast (without pCTCON plasmid) was used as a negative control under the same conditions. Each sample was analyzed in triplicate. The plate was washed with TBS-T and blocked with 3% BSA in TBS-T buffer at room temperature (RT) for 1 h. The plate was then incubated with primary influenza A M2 polyclonal antibodies (Invitrogen, USA) at a 1:3000 dilution for 2 h at RT (25°C). The wells were rinsed with TBS-T five times before adding goat anti-rabbit IgG-HRP conjugated antibodies (Invitrogen, USA) for 2 h at RT. After washing five more times, TMB substrate solutions (GenDEPOT, USA) were added for 15 min and the reactions were stopped with 1 M sulfuric acid. The absorbance of the plate was measured at 450 nm using a microplate spectrophotometer. Each sample was analyzed in triplicate and the expressions were confirmed in every batch of experiment with the same method. GraphPad Prism 8.0 software (GraphPad Software, USA) was used to analyze the results.

### Bio-panning with phage-displayed scFv libraries

The human single-fold scFv libraries, obtained from Tomlison I+J, were provided by Prof. Myung-Hee Kwon (Department of Microbiology, Ajou University School of Medicine). The libraries contain over 100 million different scFv fragments cloned in a phagemid vector pIT2, fused with His and Myc tag with ampicillin resistance, and were grown in XL1 *E*. *coli* cells (tetracycline-resistant). The phage libraries were infected with XL-1 blue *E*. *coli* cells and amplified in 2TY media with an M13K07 helper phage. The phages were precipitated using 20% PEG6000 and 2.5 M NaCl solution. Phage titers were determined using kanamycin-resistant colony forming units (CFUs) in XL-1 blue. Bio-panning methods were applied to screen M2-specific phage scFvs (M2::scFv), as previously described [36, 37]. Briefly, positive yeast (M2::YSD), blank (PBS), and negative samples (empty yeast, EBY100) diluted in PBS at an OD_600_ of 0.6 were coated onto a 96-well Maxibinding immunoplate (SPL Life Sciences, Republic of Korea) and incubated overnight in a humid box at 4°C. The plates were washed three times and blocked with a blocking buffer (3% BSA in TBS-T) for 2 h at RT. Approximately 1 × 10^9^ CFU/mL of scFv phage suspended in the blocking buffer was added to blank samples (PBS) and incubated for 2 h at RT. The non-binding scFv phages in the supernatant were transferred to a negative sample plate and incubated overnight at 4°C. The non-binding supernatant phages from negative plates were transferred to M2::YSD-coated plates (positive) for 2 h at RT. The plate was washed five times with TBS-T and dried thoroughly. Elution buffer (100 μL of 100 mM triethylamine solution) was added to each well to elute the M2::scFv phages. The eluted phages were neutralized with 1 M Tris-HCl buffer (pH 7.4) and used for the next round of bio-panning. The M2 -specific scFv selection was completed after three rounds of bio-panning.

### Isolation and production of phage display M2-specific scFv for phage ELISA

The M2::scFv selected phages were infected with XL-1 blue at an OD_600_ of 0.6 for 30 min at 37°C, without shaking, and spread on LB agar plates supplemented with tetracycline (25 μg/mL), ampicillin (100 μg/mL), and 1% glucose. The phages were incubated overnight at 37°C. Single XL-blue colonies were randomly selected from each bio-panning round and grown in 96-well plates (SPL Life Sciences, Republic of Korea). The colonies were cultured in 2TY growth media containing 25 μg/mL tetracycline, 50 μg/mL ampicillin, and 1% glucose until an OD_600_ of 0.6 was attained. The M13K07 helper phage was then added to express scFvs. The scFvs were displayed on the phage by reducing glucose concentration to 0.1% in 2TY growth media, after culturing at 30°C overnight. The collected supernatant was used as a primary antibody for the phage ELISA. Each random phage clone bound to the M2::YSD was detected using anti-M13 antibodies HRP-conjugated in a 1:1000 blocking buffer dilution (Sino biological, China) for 1 h at RT. The phage and yeast ELISA were performed according to the same protocol in following steps.

### Sequence analysis of M2-specific scFv candidates

Plasmids from each clone were extracted to detect and identify the sequences of M2-specific candidates. The individual clones of selected candidates were verified using PCR and the recommended primer pair, LMB3, 5’-CAGGAAACAGCTATGAC-3’, and pHEN, 5’-CTATGCGGCCCCATTCA-3’, under the following conditions: 95°C for 5 min, 35 cycles of 95°C for 30 s, 55°C for 30 s, 72°C for 2 min, and then 72°C for 10 min using 2X premix (Takara, Japan). The PCR products were run using 1% agarose gel electrophoresis. The plasmids with bands of approximately 900 bp or 400 bp were selected for sequencing by Macrogen (Republic of Korea). Each candidate sequence was analyzed using the IgBlast tool from NCBI [38, 39] to identify the complementarity determining regions (CDRs) and framework region (FR) of each V_H_ and V_L_.

### Engineering, production, and purification of selected M2-specific scFv candidates

One full-length scFv candidate in the phagemid pIT2 vector, NscFvM8 (720 bp), contained a stop codon in the V_H_ fragment at FR2. Therefore, to be soluble expression in BL21, site-directed mutagenesis was performed to introduce a stop codon to the glutamate residue using the Q5 site-directed mutagenesis kit (NEB, USA), according to the manufacturer’s instructions. Two different single-domain V_L_ only candidates, NVLM9 (324 bp) and NVLM10 (324 bp), were cloned in a pIg20 vector fused with protein A and PhoA leader signal peptides. Bivalent fragments (NVL2M10) were composed of two identical single domains V_L_ of NVLM10, attached by a flexible glycine-serine linker, (G_4_S)_3_. The bivalent fragment sequences (693 bp) with the *XmaI* and *NcoI* enzyme (NEB, USA) sites were synthesized and introduced into the pIg20 vector. The expressed plasmids were transformed into BL21 (DE3 pLysE) to produce soluble scFv. Protein expression was induced by adding 1 mM IPTG at an OD_600_ of 0.8. The soluble proteins in cell supernatants were collected and loaded into open columns containing Capto L resins (GE Healthcare, USA) for NscFvM8 construct, and IgG Sepharose 6 resins (GE Healthcare, USA). The eluted protein purity was confirmed by loading on SDS-PAGE with coomassie blue staining, or western blotting with primary antibodies, anti-6X His tag at a 1:3000 dilution (Abcam, UK). The ImageJ software was used to determine the purity of the purified protein.

### Influenza virions ELISA

Virus particles (1 × 10^5^ plaque-forming unit PFU/well) in PBS buffer were coated onto a Maxibinding Immunoplate (SPL Life Sciences, Republic of Korea) and incubated at 4°C overnight. The plate was washed five times with TBS-T and blocked with blocking buffer (5% skim milk in TBS-T buffer) for 2 h at RT. Then, 100 μL of the indicated proteins in a 2X serial dilution (starting from the 100 μg/well) containing blocking buffer was added and incubated at 37°C for 1 h. Polyclonal rabbit anti-HA antibodies (1:3000 dilution; Invitrogen, USA) were used as a control to ensure that the virions were present and intact for the assay. The proteins were detected using anti-6X His tag antibodies at a 1:3000 dilution (Abcam, UK) for 2 h at RT. After washing, 100 μL of a 1:5000 goat anti-mouse IgG H&L (HRP) (Abcam, UK) dilution for anti-6X His tag, and goat anti-rabbit IgG-HRP conjugated antibodies (Invitrogen, USA) for anti-HA antibodies were added and incubated for 2 h at RT. The TMB substrate and 1 M sulfuric acid were added last.

### Plaque assay and plaque reduction assay *in vitro*

MDCK cells grown in complete Eagle’s minimal essential medium (Hyclone, USA), supplemented with 10% fetal bovine serum (Gibco, USA) and 0.1% antibiotic-antimycotic (Thermo Fisher Scientific, USA), were seeded at 1 × 10^6^ cells/well in 6-well plates (SPL Life Sciences, Republic of Korea) to full confluence. H1N1/PR8 viruses with multiplicity of infection (MOI) of 0.1 were incubated with serial dilutions of NVLM10 and NVL2M10 (0, 0.1, 1, 10, and 100 μg/mL) for 24 h at 37°C. The mixture was then diluted to achieve countable plaque dilutions (approximated 0–100 PFU/well). One milliliter of the virus-protein mixture was inoculated into PBS-washed MDCK cells in duplicate. Following 1 h incubation at 37°C, the mixture was withdrawn from the cell and overlaid with 1% Seaplaque agarose containing 1 μg/mL TPCK-trypsin in DMEM. Plates were incubated at 37°C for 3 days to allow plaque formation. The cells were then fixed with 4% paraformaldehyde for 1 h and stained with 0.5% crystal violet for 30 min. Plaque assay were conducted three times with different batches of the protein expression. Plaques were counted and expressed as the percentage of PFUs.

### Immunocytochemistry (ICC)

ICC was conducted to assess the influenza HA protein in the presence of NVLM10 and NVL2M10 *in vitro*. H1N1/PR8 (MOI 0.1) was treated with 10 μg NVLM10 and NVL2M10 for 24 h, followed by infection with MDCK cells grown in 8-well chamber slides (SPL Life Sciences, Republic of Korea). Following incubation for 1 h at 37°C, the inoculum was removed and the cells were cultured in MEM containing TPCK-Trypsin (1 μg/mL) and BSA (0.2%) for 24 h. The cells were washed with PBS and fixed for 15 min using ice-cold methanol. Intracellular Staining Perm Wash Buffer (Biolegend, USA) was added for 15 min to permeabilize the cells, followed by blocking with 1% BSA and glycine in PBST buffer for 1 h. The cells were treated with polyclonal rabbit anti-HA antibodies at a 1:1000 dilution (Invitrogen, USA) for 24 h at 4°C. After washing rigorously, the cells were incubated with goat anti-rabbit IgG TRITC secondary antibodies (1:1000 dilution; Abcam, UK) for 1 h at 25°C. Vectashield Antifade mounting medium with DAPI (LSbio, USA) was added to the cells and visualized using a Zeiss LSM 700 confocal microscope. The viral protein signal was converted to relative intensity percentages using the histogram function in the Zen 3.1 (blue edition) program, by normalizing the sum of HA protein intensity (red color) to the DAPI signal (blue color) and using untreated samples as a calibrator (100%). The relative intensity percentage was measured based on the whole cells presented in the image.

### RNA extraction and one-step RTqPCR

Influenza virus A H1N1/PR8 at 2x10^4^ PFU/mL was neutralized by 10 μg for 24 h at 37°C. The mixture was then incubated to MDCK cells (MOI 0.1) for 1 h at 37°C. After removal of protein/virus complexes, the cells were cultured with MEM-free media supplemented with 0.2% BSA and TPCK-treated trypsin (1 μg/ml). The cells were harvested after 24 hpi virus challenge and stored at -20°C for further RNA extraction.

Total RNA was extracted using the TRI reagent (MRC, USA) according to the manufacturer’s instructions. The RNA concentration was determined using a spectrophotometer and diluted in distilled water (DW) at final concentration 10 ng/ μL. One-step quantitative real time PCR was performed using Accupower GreeenStar RT-qPCR Premix and Master mix (Bioneer, Republic of Korea) and Rotor-Gene Q system (Qiagen) with 50 ng of total RNA template. Data were analyzed using Rotor-Gene Q series software version 2.3.1 (Qiagen, Australia). The influenza viral genes (*HA* and *NP*) were amplified using the indicated primers (Table 1). *GAPDH* was amplified as an internal control and used for relative expression analysis.

**Table 1:** Primer list of RTqPCR analysis.

Gene name	Forward (5’– 3’)	Reverse (5’–3’)	Accession No.
*GAPDH (MDCK Cell Line)*	AACATCATCCCTGCTTCCACT	GGCAGGTCAGATCCACAAC	NM_001003142.2
*Hemagglutinin (HA)*	AGTGCCCAAAATACGTCAGG	CAGTCCATCCCCCTTCAATA	NC_002017.1
*Nucleoprotein (NP)*	CTAGCACGGTCTGCACTCAT	TCAAAGTCGTACCCACTGGC	NC_002019.1

## Results

### Isolation of M2 specific scFv/V_L_ using bio-panning

**Fig 1:**
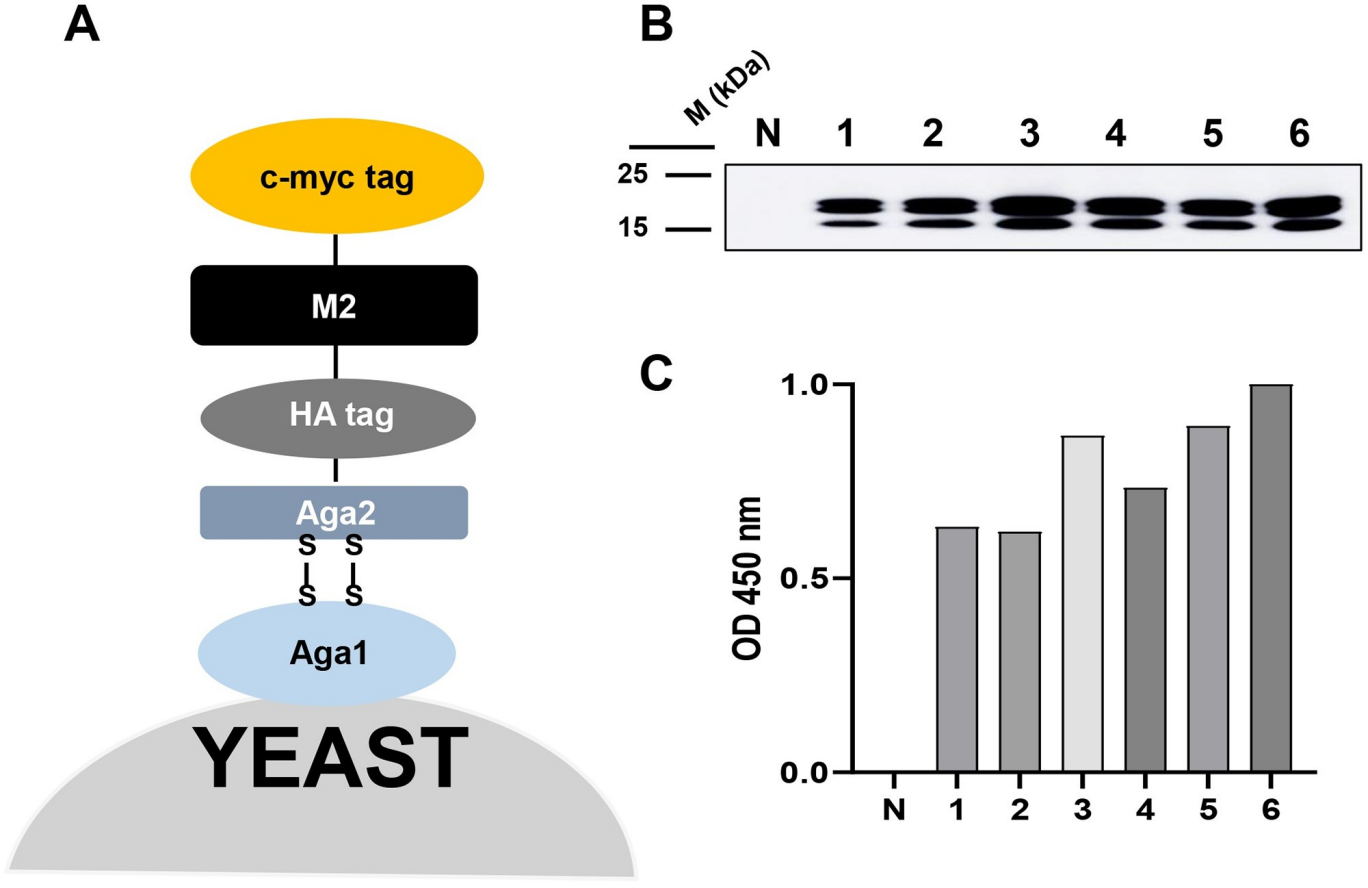
Expression of M2::YSD. (A) Diagram of YSD system. Plasmid vector M2::pCTCON containing agglutinin 2 protein (Aga2) of *Saccharomyces cerevisiae* which is naturally used to mediate yeast cell mating. The Aga2 is linked to the Aga1 protein on the cell wall by two disulfide bonds, which anchors the displayed protein assembly to the cell wall. Virus antigen was co-expressed with epitope tags (HA and cmyc) on cell surface fusion. (B) Expression of M2::YSD confirmed by WB and (C) ELISA with primary anti M2 polyclonal Abs; N, EBY100 yeast without plasmid; 1–6: 6 different expressing yeast colonies. The M2::YSD expression was confirmed in every expression batch.

**Fig 2:**
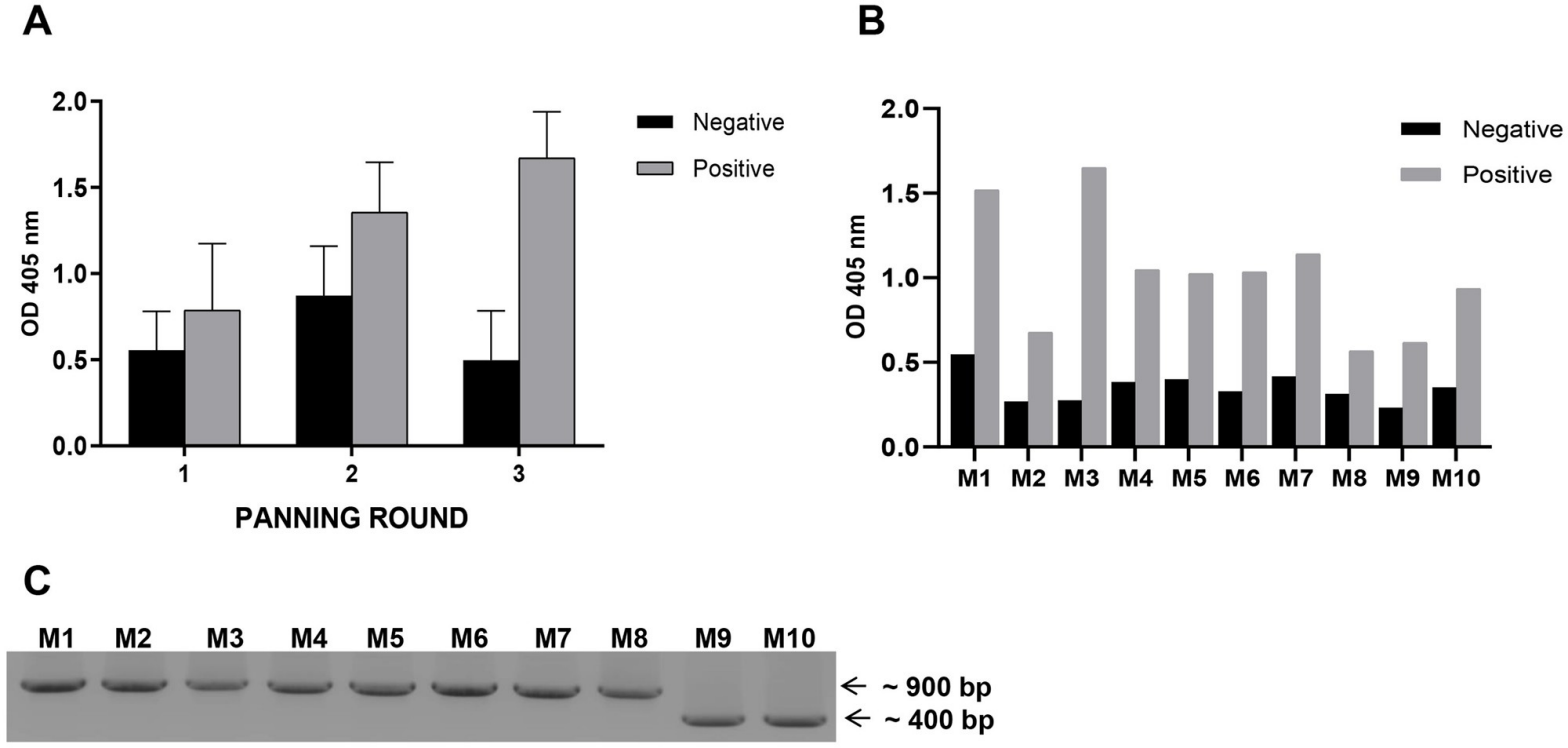
M2 specific candidates isolation using bio-panning. (A) Three rounds of bio-panning were performed with positive samples, in which M2 antigens were displayed on the yeast cell surfaces (M2::YSD), and negative samples (EBY100). Numerous phagemid colonies were randomly selected and the binding affinity measured for both positive and negative samples in each bio-panning round using Phage ELISA. Data are expressed as means ± SD. (B) Ten candidates with the highest binding affinity to M2::YSD were chosen using Phage ELISA from random phagemid colonies. (C) The 10 candidates, scFv and single-domain V_L_ forms, were detected using PCR with specific primers (scFv at 900 bp and V_L_ at 400 bp).

YSD systems were used to express antigens for bio-panning. M2 of the influenza virus A H1N1/PR8 gene sequence was synthesized (288 bp) (Accession number AY768951.1) with a change at one N-glycosylation site (N20Q) (asparagine to glutamine). The DNA fragment was amplified using PCR and introduced into a pCTCON plasmid vector for YSD (Fig 1A). M2 antigen expression in YSD was verified for six different colonies at high expression levels compared to negative controls (EBY100 yeast), using western blotting (Fig 1B) and ELISA (Fig 1C). Western blotting results indicated specific antigen expression according to size, while ELISA results exhibited the antigen expression level and the plate coating ability for bio-panning. Colonies with the highest M2 expression were selected for bio-panning. After three rounds of bio-panning, the affinity of total scFv candidates to positive samples M2::YSD was higher than the negative controls (EBY100) and this increased with each round (Fig 2A). The 10 colonies that showed the highest affinity to M2::YSD by phage ELISA were selected after three rounds (Fig 2B). These M2-specific candidates were detected using PCR with specific primer pairs. These clones were present in both the scFv form at 900 bp, and also in single-domain V_H_ or V_L_ at 400 bp (Fig 2C). The clone sequences were then analyzed to identify the complementarity determining regions (CDRs) and framework region (FR) by referring to the IgBlast Kabat antibody sequence database [40]. Unfortunately, most of the scFv clones (M1 to M7) contained a stop codon or nonsense mutation in either the CDR or FR of the V_H_ and V_L_ sequences, which are not shown in Table 1. One stop codon was identified in the V_H_-FR 2 of M8-scFv (named NscFvM8). This stop codon likely does not interfere with the phage binding affinity to M2 antigen. Two clones (M9 and M10) yielded a band at 400 bp and belonged to the V_L_ sequences. The two single-domain V_L_s exhibited highly conserved sequences in the framework region but differed in the CDR2 and CDR3 regions by only a few amino acids (Table 2).

**Table 2:** CDRs sequences of each single domain of the three candidates.

ID CLONES		CDR1	CDR2	CDR3
M8	V_H_	GFTFSSYA	ISNSGYAT	AKSTYTFDY
	V_L_	QSISSY	NAS	QQNTNSPTT
M9	V_L_	QSISSY	NAS	QQPSLPPPT
M10	V_L_	QSISSY	LAS	QQQGPHPTT

### M2-scFv/V_L_ characterization and engineering to increase affinity

**Fig 3:**
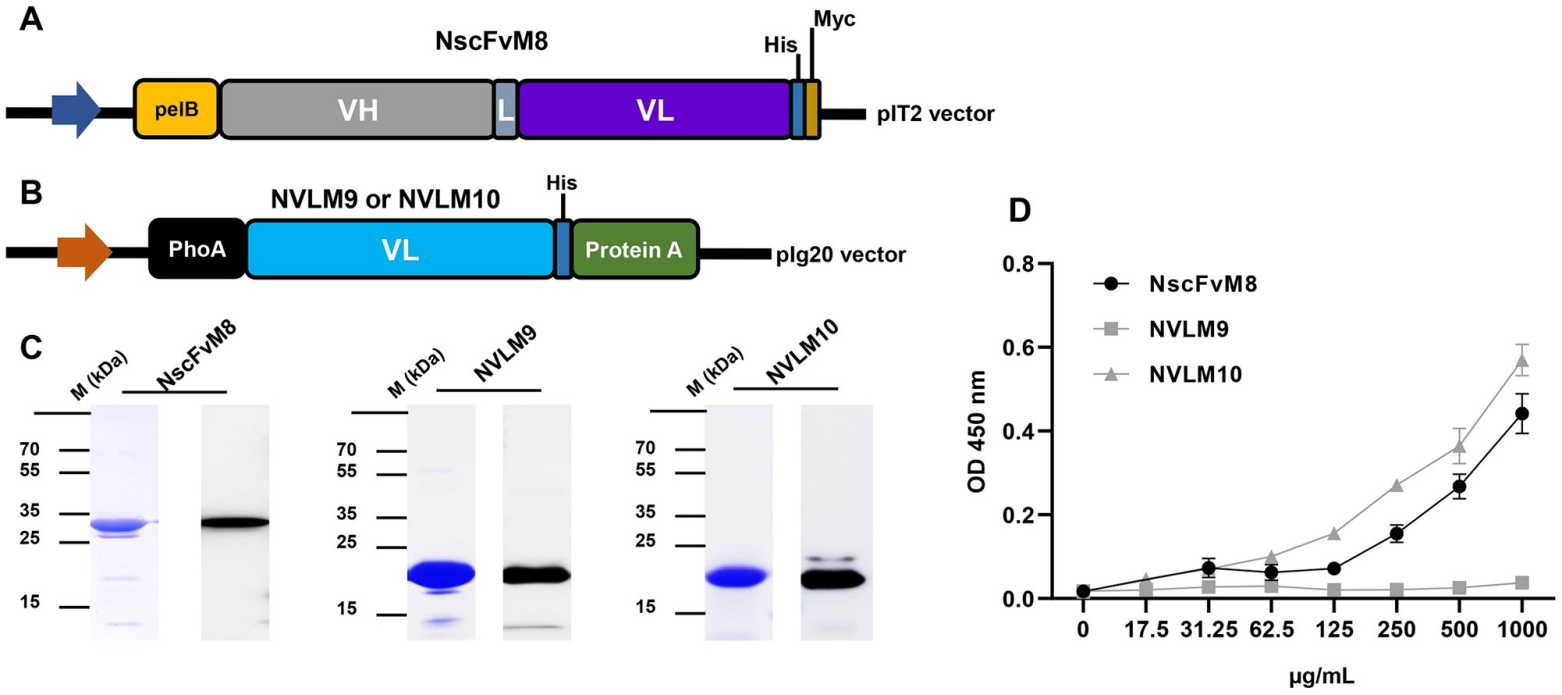
Production of selected candidates in *E*. *coli* and their respective virion binding affinities. (A) Expression vector of a single-chain variable fragment candidate, NscFvM8, in a pIT2 vector fused with pelB at the N terminal, and a His, cmyc tag at the C terminal. (B) Expression vector of 2 different single-domain V_L_ candidates, NVLM9 and MVLM10. The single-domain V_L_ genes were amplified and inserted into the pIg20 vector containing a PhoA signal peptide at the N terminal and fused with protein A to improve purification. (C) Purification results of three candidates confirmed by coomassie blue staining and western blotting using anti-His-Abs, with the size of each protein indicated, left, NscFvM8; middle, NVLM9; right, NVLM10. (D) Virion ELISA results showing purified candidates bound to virus particles in a concentration-dependent manner. The background was subtracted.

To characterize candidates at the protein level, two single-domain V_L_ clones, NVLM9 and NVLM10, were constructed with a pIg20 vector containing PhoA signal peptide and protein A to ease expression and purification. For the NscFvM8 candidate, site-directed mutagenesis changed the stop codon to a glutamate residue, similar to the phagemid vector that contained a stop codon. The NscFvM8 sequences fused with His and Myc tag at the C-terminal in the pIT2 vector were expressed in the *E*. *coli* BL21 (DE3) pLysE strain (Fig 3A and 3B). All three candidates were secreted into the media containing a signal peptide (PhoA or pelB). The expressed proteins were purified and verified using coomassie blue staining and western blotting with anti-6X His tag antibodies, which were tagged to the expression vector. The proteins, NscFvM8, NVLM9, and NVLM10, were successfully purified and displayed on the gel at 30.1, 22.5, and 22.5 kDa, respectively (Fig 3C). However, the yield and purity of each protein differed. NscFvM8 was obtained at 84% purity with a 0.5 mg/L yield, NVLM9 at 91% purity and a 3 mg/L yield, and NVLM10 at 99% purity with a 3 mg/L yield (Table 3).

**Table 3:** Characterization of three candidates with purification yield.

Protein	Form	Size (kDa)	Expression	Yield (mg/L)	Purify (%)
NscFvM8	V_H_-L-V_L_	30.1	Secret	0.5	84
NVLM9	V_L_	22.5	Secret	3	91
NVLM10	V_L_	22.5	Secret	3	99

**Fig 4:**
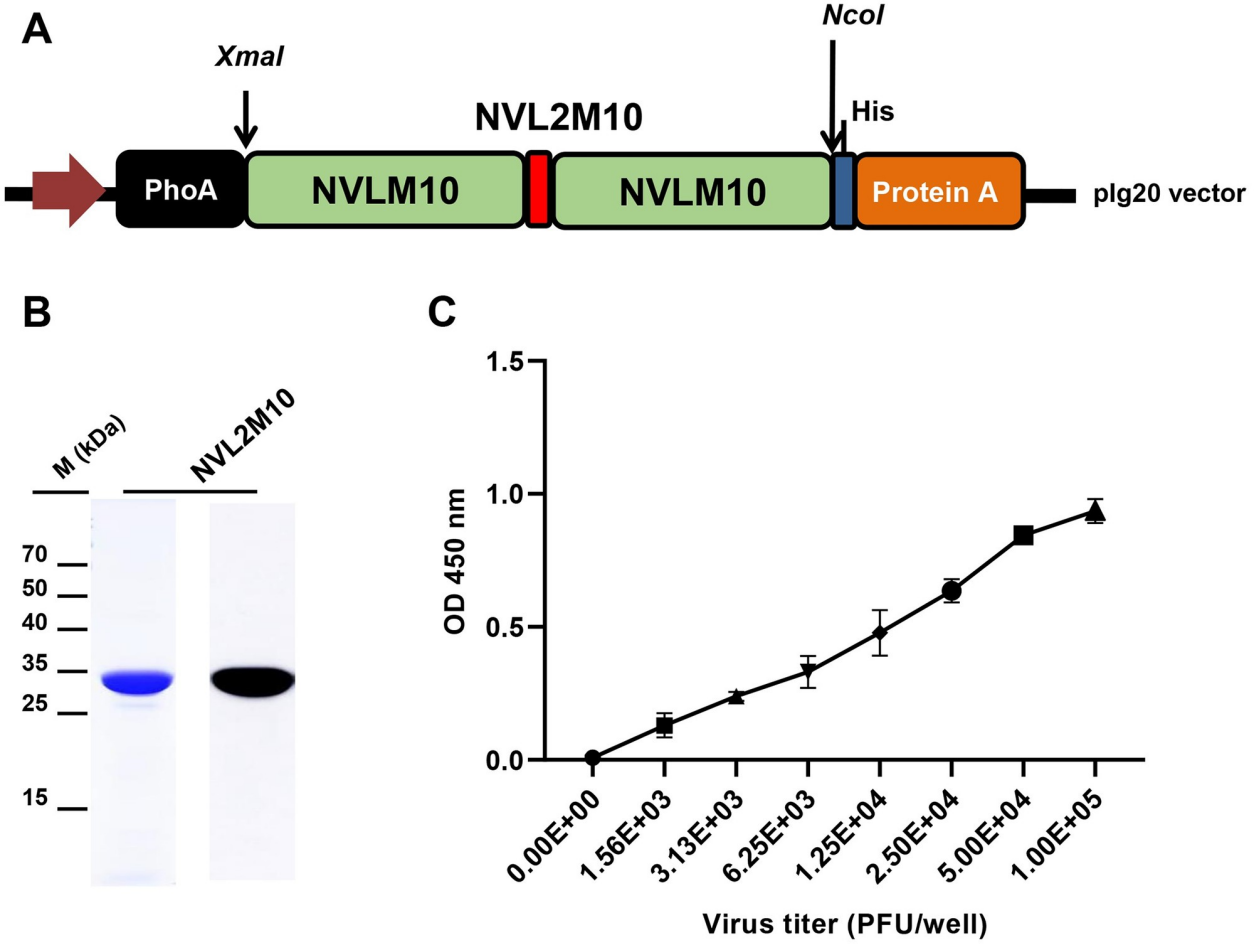
Engineering of a single-domain V_L_ antigen specific to the M2 protein of the H1N1/PR8 virus to a bivalent form. (A) A bivalent single-domain V_L_ specific to M2 (NVL2M10) was constructed by linking two identical single-domain V_L_s with a linker (G_4_S)_3_ and introducing it into the expression vector pIg20. (B) Purification of NVL2M10 (35 kDa) using Sepharose IgG resin exhibited high purity with coomassie blue staining and could detect anti His Abs in western blotting. (C) The binding affinity of NVL2M10 to influenza virions. A total of 250 μg/mL of NVL2M10 bound to coated influenza virus particles at a 2X serial dilution. The background was subtracted.

The direct antigen binding affinity to the influenza virus particle H1N1/PR8 of the functional recombinant protein was assessed through indirect ELISA with a 2X serial dilution. NVLM9 exhibited no affinity to the H1N1/PR8 virus particles. NVLM10 and NscFvM8 exhibited concentration-dependent affinities. However, NVLM10 presented the highest interaction with H1N1/PR8, even at an OD_450_ value of approximately 0.5 (Fig 3D). NscFvM8 also showed affinity to the influenza virions, however, the proteins were obtained at a low concentration (0.5 mg/L) and purity. Therefore, NVLM10 was chosen to engineer into a bivalent form with a linker to increase the candidate binding affinity to the virus, which is critical for antiviral activity against H1N1/PR8 (Fig 4A). The bivalent NVL2M10 was expressed in a secreted form in BL21 (DE3) pLysE strains. It was also further purified and verified with coomassie blue staining and detected using western blotting (Fig 4B) at a high yield of 3.5 mg/L with 98% purity.

**Fig 5:**
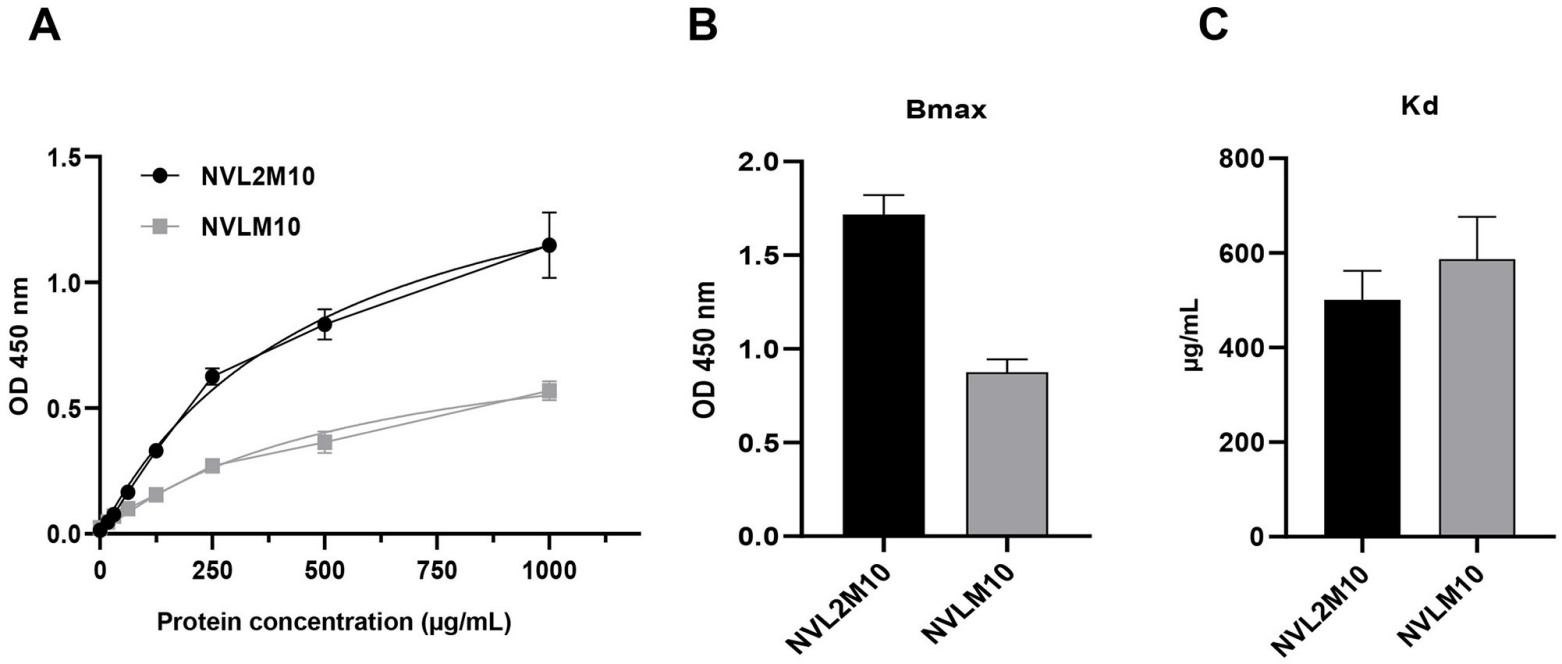
The increasing virus particle binding affinity of the bivalent form compared to monovalent single-domain V_L_. (A) Influenza A virions were used as the antigen and coated for ELISA. A 2X serial dilution of the NVLM10 and NVL2M10 proteins with starting amounts of 100 μg/well (1000 μg/mL) were added for binding activity. (B), (C). B_max_ and K_D_ values were calculated using site-specific binding with a nonlinear best-fit regression model in GraphPad Prism 8.0.

As expected, the bivalent protein (250 μg/mL) demonstrated a high affinity for different densities of the virus H1N1/PR8 coated through the virion ELISA method. The virion ELISA sensitivity was approximately 1.25 × 10^4^ PFU of the virus when using 250 μg/mL of protein (Fig 4C). Virion ELISA was performed to compare the binding kinetics of serial dilutions of the same amount of the two proteins. The bivalent NVL2M10 had a higher virion binding affinity than monomeric NVLM10 at each amount (Fig 5A). Single-domain NVLM10 presented a maximum viral particle binding (B_max_) of 2X lower than that of bivalent NVL2M10 (Fig 5B). However, the equilibrium dissociation constant (K_D_) value of NVL2M10 (500 μg/mL) was not significantly different from that of NVLM10 (580 μg/mL).

### Neutralization activity of single-domain NVLM10 and bivalent NVL2M10 against virus H1N1/PR8 *in vitro*

**Fig 6:**
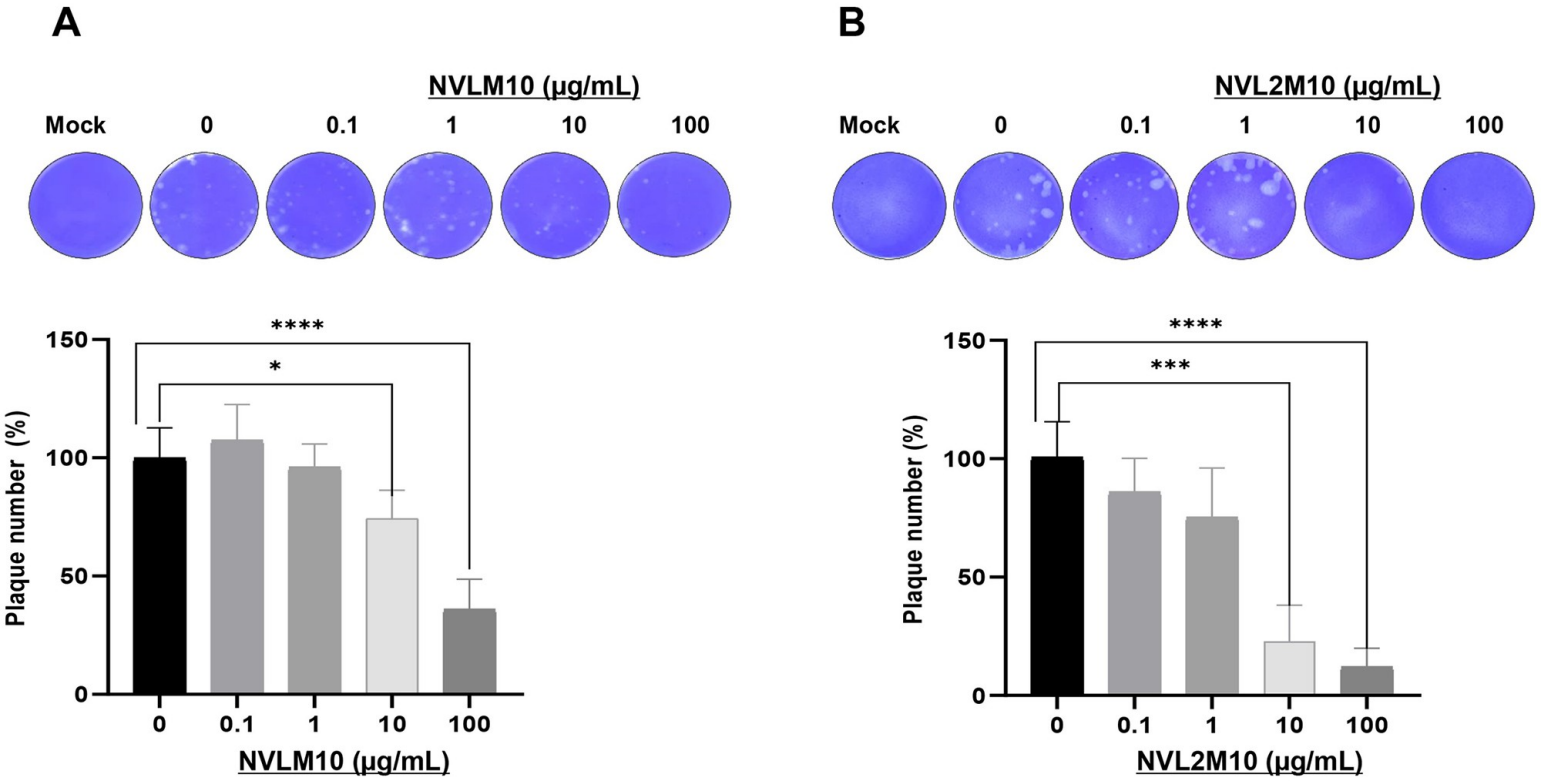
Neutralization activity to influenza virus infection of the NVLM10 and NVL2M10 *in vitro*. Influenza H1N1/PR8 virus infection in MDCK cells was neutralized in a treatment of serially diluted concentrations (0, 0.1, 1, 10, 100 μg/mL) of (A) NVLM10 or (B) NVL2M10 through plaque inhibition assay. Plaques were counted and represented as a plaque number percentage. Statistical significance was determined by unpaired t test (*p < 0.05, ***p < 0.0005, ****p < 0.0001).

The virion binding activity of NVL2M10 and NVLM10 promoted neutralizing activity that prevented influenza virus replication *in vitro*. A plaque inhibition assay was performed to identify whether two different M2-specific V_L_s (NVLM10 and NVL2M10) each neutralized virus infection. Purified NVLM10 and NVL2M10 at a 10X serial dilution were preincubated with H1N1/PR8 viruses. MDCK cells were infected with the protein/virus mixtures to perform the plaque reduction assay. The plaque numbers were visibly reduced in the presence of NVLM10 and NVL2M10 in a dose-dependent manner (Fig 6). Plaques were significantly reduced to 27% at 100 μg/mL NVLM10 (Fig 6A), 10% at 10 μg/mL, and 5% at 100 μg/mL NVL2M10 (Fig 6B).

**Fig 7:**
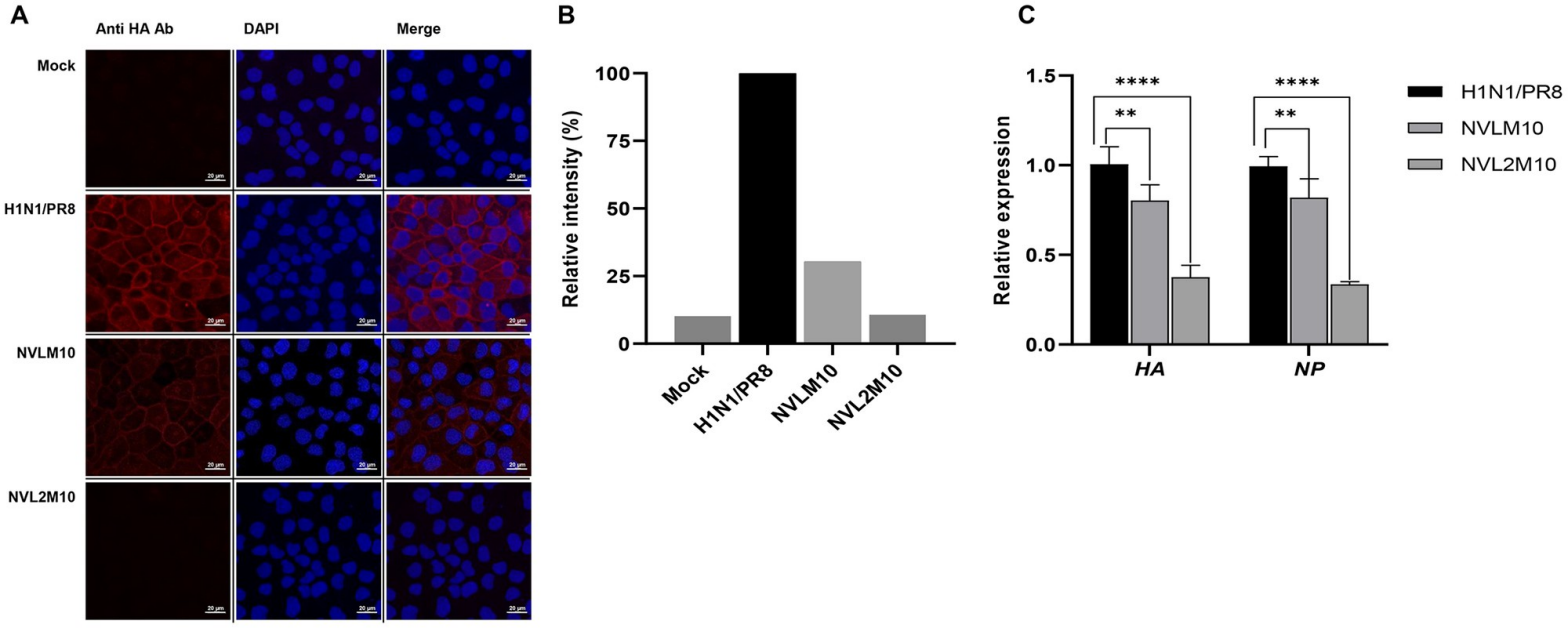
*In vitro* reductions of viral proteins and viral genome by NVLM10 and NVL2M10. (A) Visualization of HA protein expression in H1N1/PR8 (MOI 0.1) infected MDCK cells in the presence of 10 μg each of NVLM10 and NVL2M10 compared to H1N1/PR8-infected and mock cells at 24 hpi (magnification 40X). The HA proteins of influenza H1N1/PR8 were detected using the primary polyclonal rabbit anti-HA antibodies. (B) The viral protein signal was converted to relative intensity percentages using the histogram function in Zen 3.1 (blue edition) program, by normalizing to the DAPI signal and using untreated samples as a calibrator. (C) At 24 hpi, H1N1/PR8 infected MDCK cells were collected and processed for RTqPCR. The relative expression of viral gene HA and NP was quantified to compare H1N1/PR8 with NVLM10 and NVL2M10 (treated) to that of H1N1/PR8 untreated group. Unpaired t test were used to determine p value (**p < 0.005, ****p < 0.0001).

Next, viral HA protein reductions caused by the neutralizing activity of the two proteins were measured via immunocytochemistry to determine the efficiency differences between NVL2M10 and NVLM10. Viruses neutralized with 10 μg of each protein were inoculated into MDCK cells at an MOI of 0.1. Infection proceeded for 24 h and the HA protein of the influenza virus was detected using primary polyclonal rabbit anti-HA antibodies and traced using TRITC (red). NVL2M10 inhibited virus growth, which was confirmed by the absence of a red signal. However, HA proteins were detected at a lower intensity with 10 μg NVLM10 treatment (Fig 7A). The relative intensity percentages were calculated and expressed in graphs (Fig 7B). The 10 μg NVLM10 treatment could not prevent viral infection completely. HA protein intensity dropped from 100% to 30%, whereas NVL2M10 decreased it by 10%. Consistently, NVLM10 and NVL2M10 decreased *HA* and *NP* gene expression level at similar ratio (Fig 7C). These results demonstrate the neutralizing activity of NVLM10 and NVL2M10 on influenza H1N1/PR8.

## Discussion

Single-domain antibodies (sdAbs) (V_H_ or V_L_) have the simplest forms that can retain antigen binding among Fab and scFv Abs. Approximately 61 sdAbs target the respective antigens with potential therapeutic applications [10]. SdAbs have been engineered to enhance solubility, specificity, and affinity [41–43]. The M2 protein is a main target for the development of viral drugs and universal vaccines against influenza infections. M2-specific monoclonal antibodies and monoclonal scFvs are also protective against influenza infection *in vitro* and in animal models [32, 44–47]. However, these specific mAbs showed no- or partial neutralization activity. The main mechanisms are dependent on antibody-dependent cell-mediated cytotoxicity, complement-dependent cytotoxicity, and antibody-dependent cell-mediated phagocytosis [22, 24, 28]. A synthetic camel-derived single-domain antibody (V_H_H) reportedly neutralized the influenza A virus by specifically binding to the M2 protein [48]. In the present study, we selected antigen M2-specific candidates from human scFv libraries using bio-panning. The single-domain V_L_ Abs, NVLM10, and its engineered bivalent molecule, NVL2M10, exhibited neutralization activity against H1N1/PR8 with plaque and viral protein, viral genome expression reductions *in vitro* by binding to influenza virions.

YSD systems were used to express the M2 protein antigen in bio-panning. YSD is used to engineer proteins for therapeutic, diagnostic, and biotechnology applications because of its high protein expression capabilities, eukaryotic post-translational machinery, and the use of fluorescence-activated cell sorting (FACS) [49, 50]. Furthermore, M2 proteins anchored to the yeast cell wall resemble the M2 proteins on the influenza virus surface, supporting specific selection and increasing binding efficiency to the native antigen form. However, yeast glycosylation limits viral protein expression, which was overcome by changing one amino acid at the N-glycosylation site (N20Q) in the M2 protein sequences from asparagine to glutamine. Consequently, the antigen M2 protein was efficiently expressed in the YSD system and prepared for bio-panning (Fig 1). Human scFv fragments obtained from a single human framework in the Tomlinson I+J libraries were used to screen the M2 specific binders using bio-panning. Three rounds of bio-panning were performed to isolate several candidates with high affinity for M2::YSD (Fig 2). Unfortunately, the high negative selection from each round was noted. Generally, phage ELISA was performed to select the candidates that exhibited higher affinity to M2::YSD (positive) compared to EBY yeast (negative). The scFvs were displayed on M13K07 helper phage surface and detected indirectly by the use of anti-M13 antibodies HRP-conjugated which might result in the unspecific binding. Importantly, the background of yeast cells coated in the plates (OD600 0.4–0.6) expressed relatively high values. Therefore a hundred random colonies could be shown by biopanning screening and a few candidates showing the differences as a positive colonies were screened. To select more positive colonies, we tried to screen many samples (blank plate, yeast cell). These candidates were further determined using PCR and the gene sequences analyzed for IgBlast. Two of the three clones were produced at high yields in *E*. *coli*. The single-chain variable fragment (NscFvM8), consisting of V_H_ and V_L_ domains, was expected to show a higher affinity to virus particles than single-domain V_L_ (NVLM9 and NVLM10). However, the virion ELISA indicated that single-domain NVLM10 had the highest affinity. Moreover, the NVLM9 single-domain did not exhibit binding affinity to the influenza virus particles (Fig 3D). The differences in the first CDR2 amino acid and a few CDR3 amino acids of the two V_L_ fragments distinguished the antigen binding (Table 1). Because NVLM10 exhibited the highest influenza virion affinity and efficient production with high purity in *E*. *coli*, we decided to engineer the protein into bivalent single-domain fragments, to produce NVL2M10. Up to 500-fold increases in bivalent anti-TNF sdAbs (V_H_H) were reported with rheumatoid arthritis treatment *in vivo*, compared to monovalent molecules [51]. Therefore, we hypothesized that a bivalent format of the single-domain V_L_ could enhance the antigen-binding affinity, at least 2-fold, compared to the monomer form. The bivalent protein, NVL2M10, was produced by connecting two identical single-domain V_L_s (M10) through a flexible linker that is commonly used in scFv structures. The protein was obtained in a soluble form with high yield and exhibited binding affinities to different virus particle concentrations (Fig 4). Compared to single-domain V_L_, NVLM10, the bivalent NVL2M10 displayed an increased binding affinity for the influenza virions. Maximum binding (B_max_) is the maximum binding affinity of the antibody to the constant antigen. The equilibrium dissociation constant (K_D_) is defined by the antibody concentration that exhibits 50% antigen binding affinity [32]. Therefore, antigen binding efficiencies were determined with the expectation of higher B_max_ values and lower K_D_ values. The B_max_ value of NVL2M10 at OD_450_ (optical density) was 2X greater than that of NVLM10, while the K_D_ value of NVL2M10 was slightly lower than that of NVLM10 (Fig 5). While the typical K_D_ value of a commercial antibody is 1 nM [52], the K_D_ value of several antibodies targeting the M2 protein is 0.412 nM for full-length antibodies (14C2), 39.5 nM for VhHs [48], or lower than 4 μg/mL for several others [32]. Unfortunately, our candidates had noticeably higher K_D_ values, implying that longer timer or higher temperatures were required to enable tight binding to the influenza virus particles. Furthermore, the presence of a small proportion of M2 proteins, 16–67 molecules per 500 HA molecules per virion [53] and the smaller size of M2e (23 aa), possibly explains the low binding efficiency of NVLM10 and NVL2M10. Despite the low binding affinity to virus particles, only 100 μg/mL of NVLM10 inhibited viral infection with an 80% reduction in plaque number and HA protein *in vitro* (Figs 6A and 7*)*. The bivalent single-domain NVL2M10 was designed using NVLM10 molecules to improve binding efficiency, resulting in a 2X higher B_max_ value (Fig 5B). Notably, a 10X reduction of NVL2M10 to 10 μg increased neutralization activity to 90% inhibition of viral infection.

Taken together, our findings potentially provide an additional approach for the development of neutralizing antibodies against influenza viruses using the single-domain V_L_ targeting M2 protein. However, the viral infection mechanisms of these candidates require further investigation. Further studies on the improvement of virion binding efficiency to enhance neutralizing activity are also necessary.

## Supporting information

S1 Raw images (PDF)
