# Supplementary material for: Enhancing neutralizing activity against influenza H1N1/PR8 by engineering a single-domain VL-M2 specific into a bivalent form
Source: PLoS One. 2022 Aug 31;17(8):e0273934. doi: 10.1371/journal.pone.0273934 (PMC9432714; doi:10.1371/journal.pone.0273934)

Fig 1. Expression of M2::YSD

(B) Expression of M2::YSD confirmed by WB with primary anti M2 polyclonal Abs;

N, EBY100 yeast without plasmid; 1-6: 6 different expressing yeast colonies.

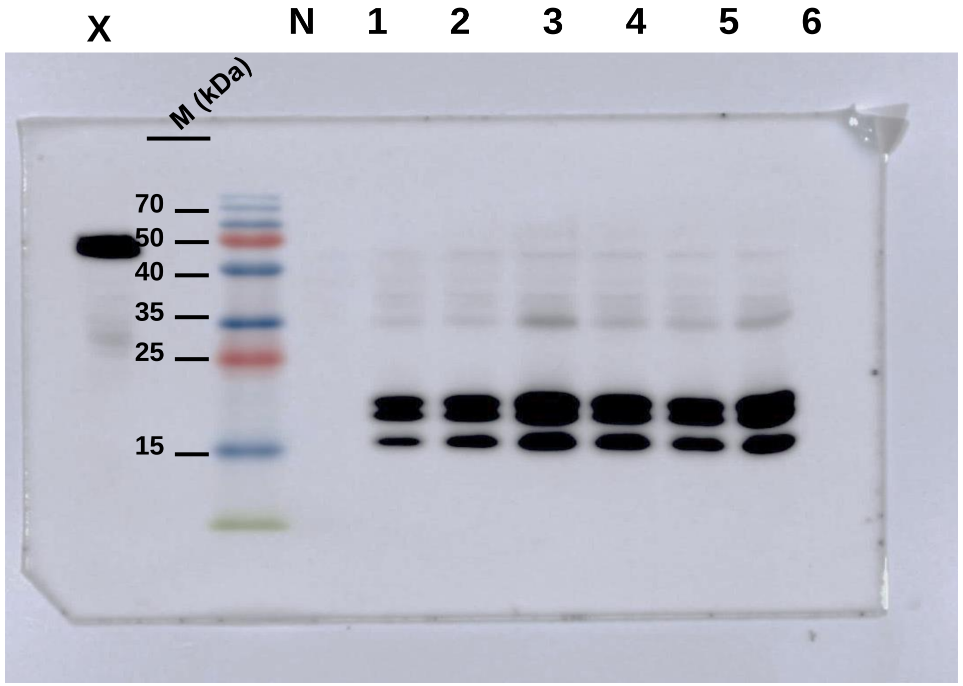

Fig 2. M2 specific candidates isolation using bio-panning.

(C) The 10 candidates, scFv and single-domain V_L forms, were detected using PCR with specific primers (scFv at 900 bp and V_L at 400 bp) were loaded to agarose gel.

C

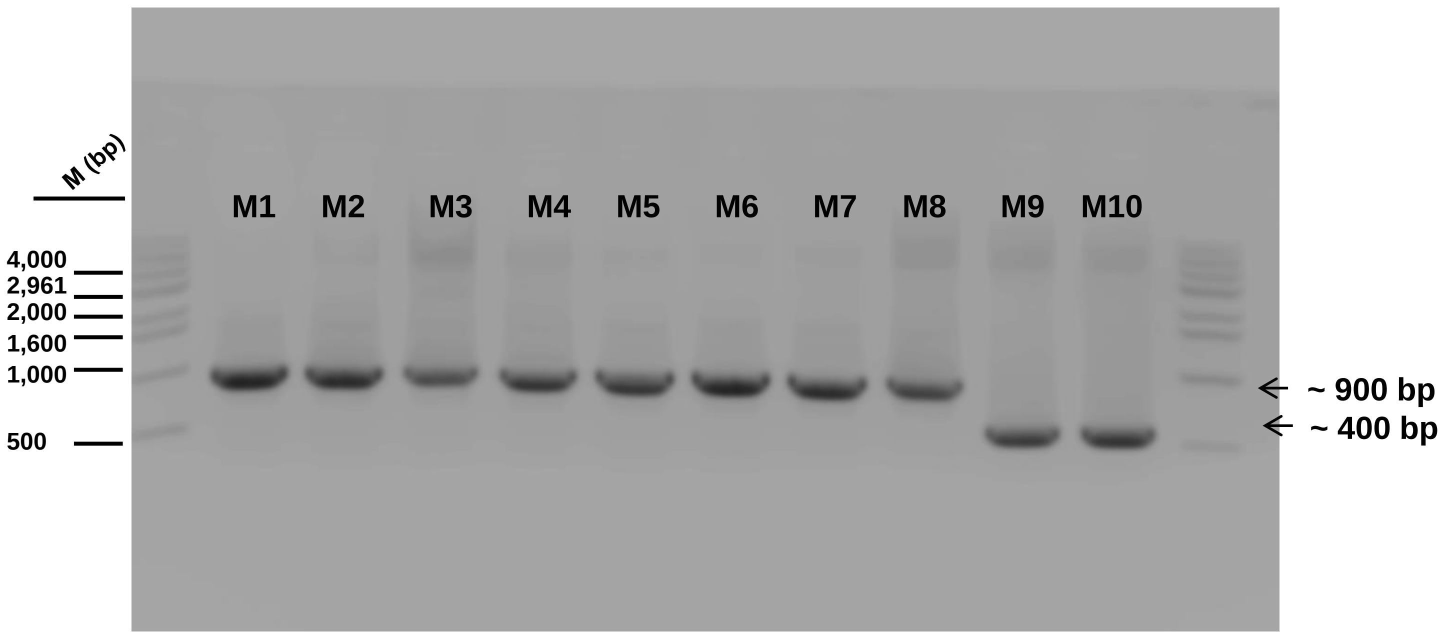

Fig 3. Production of selected candidates in *E. coli* and their respective virion binding affinities.

(C) Purification results of three candidates confirmed by coomassie blue staining and western blotting using anti-His-Abs, with the size of each protein indicated, left, **NscFvM8**;

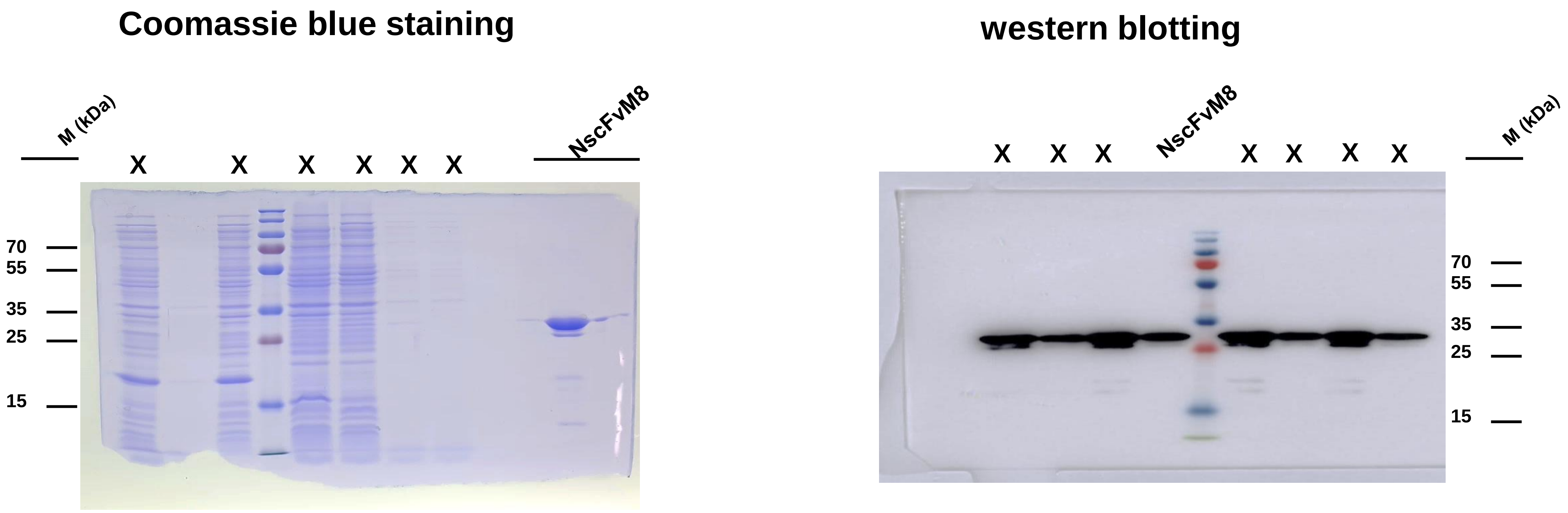

Fig 3. Production of selected candidates in *E. coli* and their respective virion binding affinities.

(C) Purification results of three candidates confirmed by coomassie blue staining and western blotting using anti-His-Abs, with the size of each protein indicated, middle, **NVLM9**

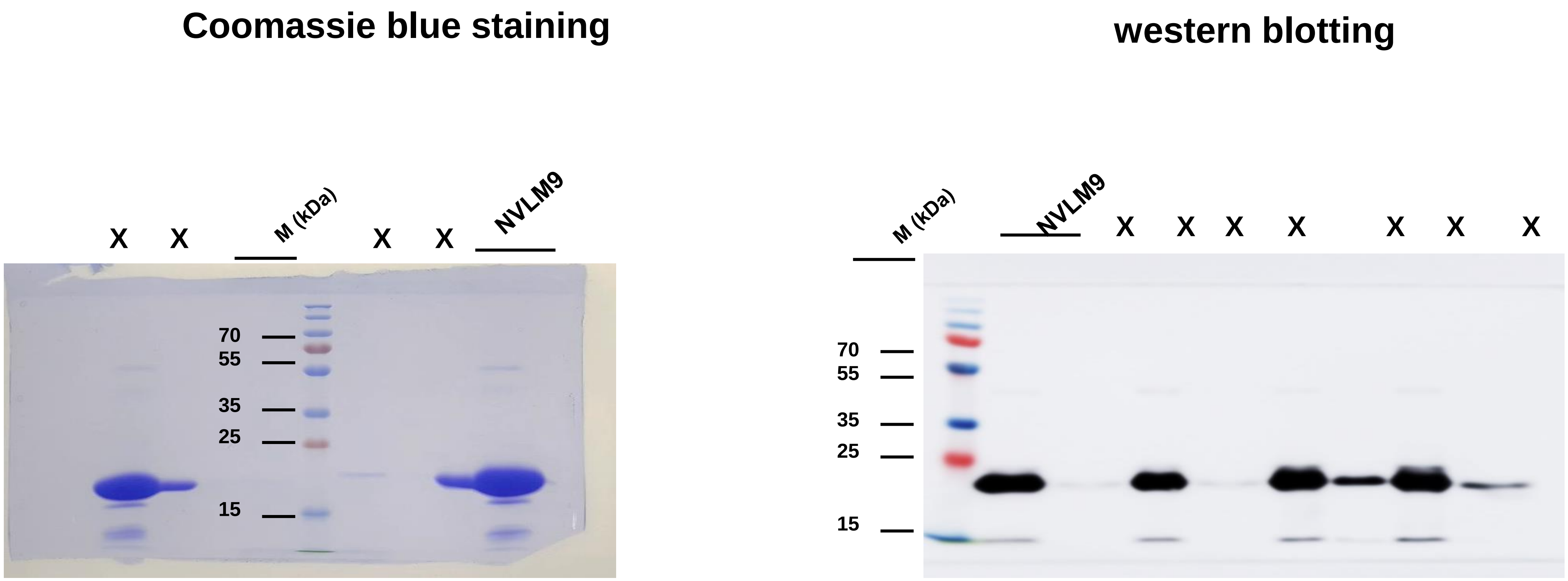

Fig 3. Production of selected candidates in *E. coli* and their respective virion binding affinities.

(C) Purification results of three candidates confirmed by coomassie blue staining and western blotting using anti-His-Abs, with the size of each protein indicated, right, **NVLM10.**

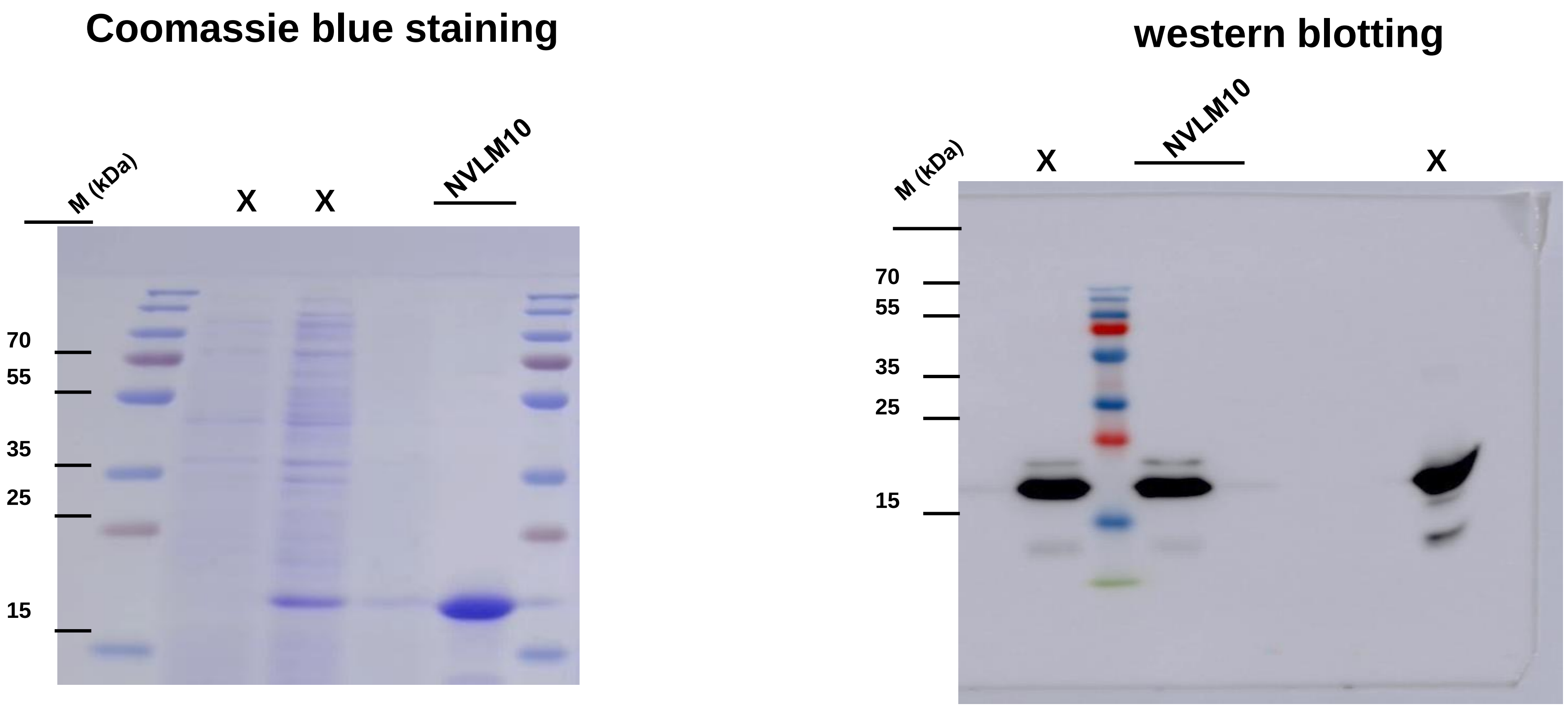

Fig 4. Engineering of a single-domain V_L antigen specific to the M2 protein of the H1N1/PR8 virus to a bivalent form.

(B) Purification of NVL2M10 (35 kDa) using Sepharose IgG resin exhibited high purity with coomassie blue staining and could detect anti His Abs in western blotting.

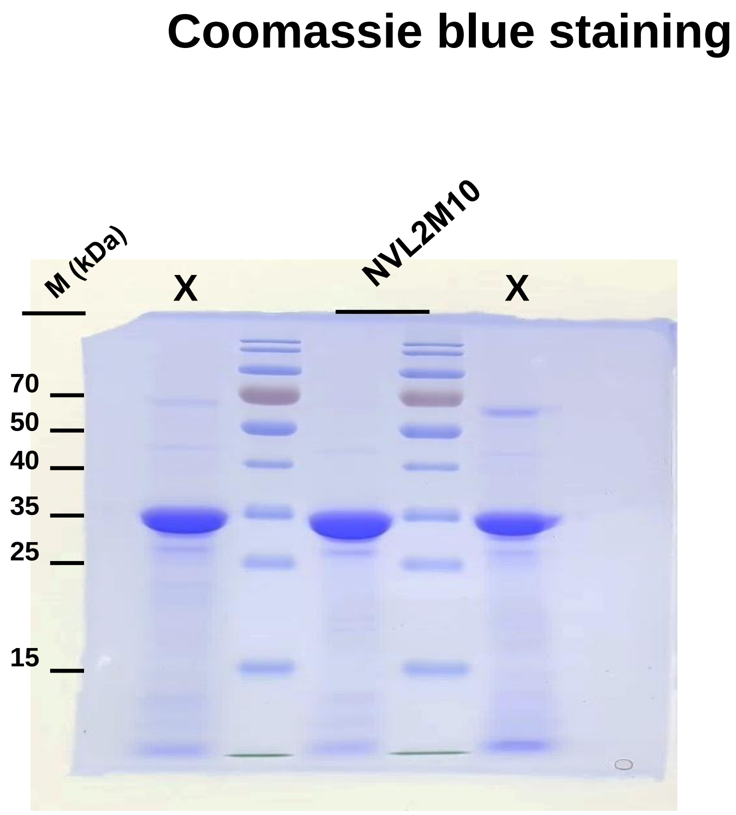

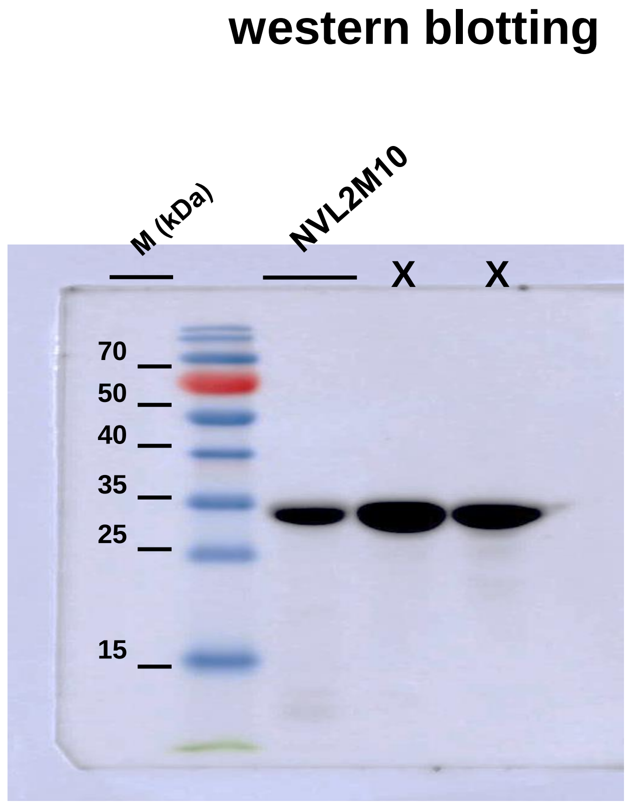

Supplement: S1 Raw images — (PDF) [file pone.0273934.s001.pdf]
